# A Necessary Role for Increased Biglycan Expression during L1-Mediated Colon Cancer Progression

**DOI:** 10.3390/ijms23010445

**Published:** 2021-12-31

**Authors:** Arka Saha, Sanith Cheriyamundath, Anmol Kumar, Nancy Gavert, Thomas Brabletz, Avri Ben-Ze’ev

**Affiliations:** 1 Department of Molecular Cell Biology, Weizmann Institute of Science, Rehovot 7610001, Israel; arka.saha@weizmann.ac.il (A.S.); sanith.cheriyamundath@weizmann.ac.il (S.C.); anmol.kumar@atmiyauni.ac.in (A.K.); nancy.gavert@weizmann.ac.il (N.G.); 2 Department of Experimental Medicine I, Nikolaus-Feibiger-Center for Molecular Medicine, University of Erlangen-Nuernberg, 91054 Erlangen, Germany; thomas.brabletz@fau.de

**Keywords:** biglycan, colorectal cancer (CRC), L1CAM (L1), NF-κB

## Abstract

Aberrant activation of Wnt/β-catenin signaling and downstream β-catenin-TCF target genes is a hallmark of colorectal cancer (CRC) development. We identified the immunoglobulin-like cell adhesion receptor L1CAM (L1) as a target of β-catenin-TCF transactivation in CRC cells. Overexpression of L1 in CRC cells confers enhanced proliferation, motility, tumorigenesis, and liver metastasis, and L1 is exclusively localized at invasive areas of human CRC tissue. Several genes are induced after L1 transfection into CRC cells by a mechanism involving the L1-ezrin-NF-κB pathway. We conducted a secretomic analysis of the proteins in the culture medium of L1-overexpressing CRC cells. We detected a highly increased level of biglycan, a small leucine-rich ECM component, and a signaling molecule. We found that induction of biglycan is required for the cellular processes conferred by L1, including enhanced proliferation, motility, tumorigenesis, and liver metastasis. The suppression of endogenous biglycan levels or a point mutation in the L1 ectodomain that regulates cell–cell adhesion mediated by L1 blocked the enhanced tumorigenic properties conferred by L1. The mechanism of biglycan induction by L1 involves the L1-NF-κB pathway. Blocking NF-κB signaling in L1 expressing cells suppressed the induction of biglycan and the tumorigenic properties conferred by L1. Biglycan expression was undetectable in the normal colonic mucosa, but expressed at highly increased levels in the tumor tissue, especially in the stroma. The therapeutic strategies to target biglycan expression might provide a useful approach for CRC treatment in L1-overexpressing tumors.

## 1. Introduction

Aberrant activation of the Wnt/β-catenin pathway and its downstream β-catenin-T cell factor (TCF) target genes is a characteristic feature of colorectal cancer (CRC) development [1]. Among such targets of Wnt signaling, we detected the immunoglobulin-like cell adhesion receptors L1CAM (L1) and Nr-CAM [2,3]. Overexpression of L1 in CRC cells confers enhanced proliferation, motility, tumorigenesis, and liver metastasis [2,4]. Moreover, L1 is expressed in CRC cells at the invasive front of human CRC tissue, but not in adenomas and homeostatic colonic mucosa [2]. The signaling by L1 in CRC cells involves an L1-ezrin-NF-κB pathway [5,6] and results in the activation of numerous genes that contribute to the tumorigenic properties conferred by L1 overexpression [6,7,8,9,10,11,12,13,14], including several genes defined as colonic stem cell signature genes [6,8,10,11]. Aiming to characterize L1-induced genes required for conferring tumorigenic properties in CRC cells, we have analyzed the secretome of L1-overexpressing cells and detected several proteins whose levels are dramatically enhanced in L1-expressing CRC cells [9,14]. In this study, we investigated the role of the small, leucine-rich, proteoglycan biglycan that is both an extracellular matrix (ECM) component, but can also act in cell signaling using multiple signaling pathways [15], and is implicated in the tumorigenesis of different types of cancer [16]. We addressed the involvement of biglycan in L1-mediated CRC progression.

## 2. Results

### 2.1. Induction of Biglycan Levels in the Secretome and in CRC Cells Overexpressing L1

**Figure 1:**
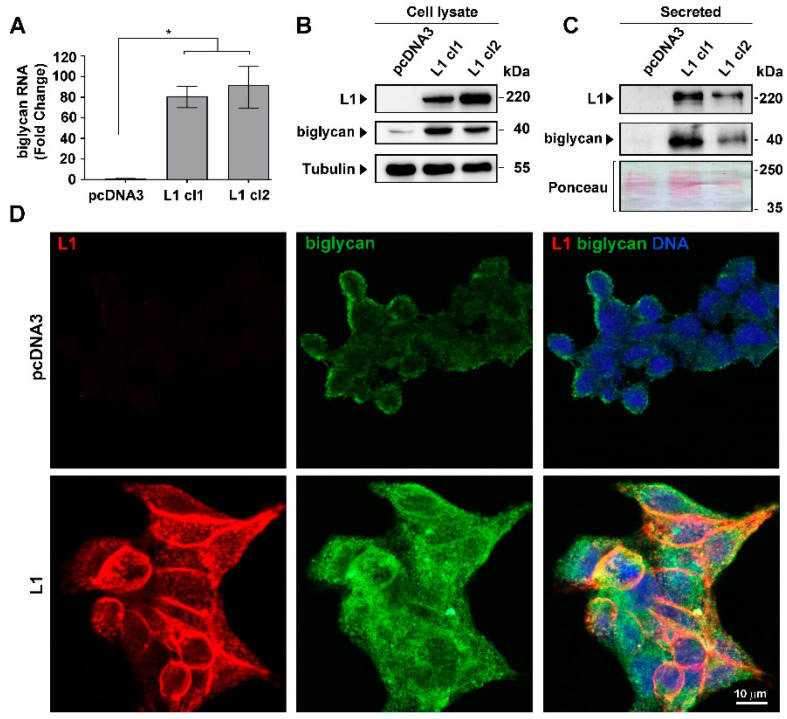
Induction of biglycan expression in CRC cells by L1. (**A**) The increased expression of biglycan RNA in clones of LS 174T CRC cells transfected with L1 (L1 cl1 and cl2) was compared to control pcDNA3-transfected cells. (**B**) Western blotting determined biglycan protein levels in the LS 174T clones described in (**A**). (**C**) The levels of secreted biglycan in the culture medium of LS174T cells expressing L1 were compared to those in control pcDNA3-transfected cells. (**D**) Double immunofluorescence labeling for L1 and biglycan in LS 174T cells expressing L1 and in control pcDNA3 transfected CRC cells. The nuclei were stained with DAPI. * *p* < 0.05.

To identify proteins secreted by L1-expressing CRC cells whose levels are increased after L1 transfection, we performed mass spectrometry analysis on proteins in the secretome of LS 174T L1-expressing and control LS 174T cells. As shown in Supplementary Table S1, the levels of a number of proteins increased dramatically in the secretome of L1-overexpressing CRC cells. Since changes in ECM proteins and their organization around the tumor tissue are a hallmark of cancer progression, we turned to investigate the small leucine-rich ECM protein biglycan whose levels in the secretome increased manyfold upon L1 transfection (Supplementary Table S1). L1-expressing CRC cell clones displayed a significant increase in both biglycan RNA and protein levels compared to control, empty vector-transfected CRC cells (Figure 1A,B). In addition, as expected from the data shown in Supplementary Table S1, we found that the culture medium from L1-expressing cells contained a much higher level of biglycan than control cells (Figure 1C). Moreover, while control, empty-vector transfected cells displayed a weak pericellular distribution of biglycan, upon L1-transfection the cells showed a dramatically higher level of endogenous biglycan (Figure 1D). These results suggest that overexpression of L1 in LS 174T CRC cells results in increased expression and secretion of biglycan from CRC cells.

### 2.2. Changes in Biglycan Expression Affect the Growth Rate and Motility of L1-Expressing CRC Cells

**Figure 2:**
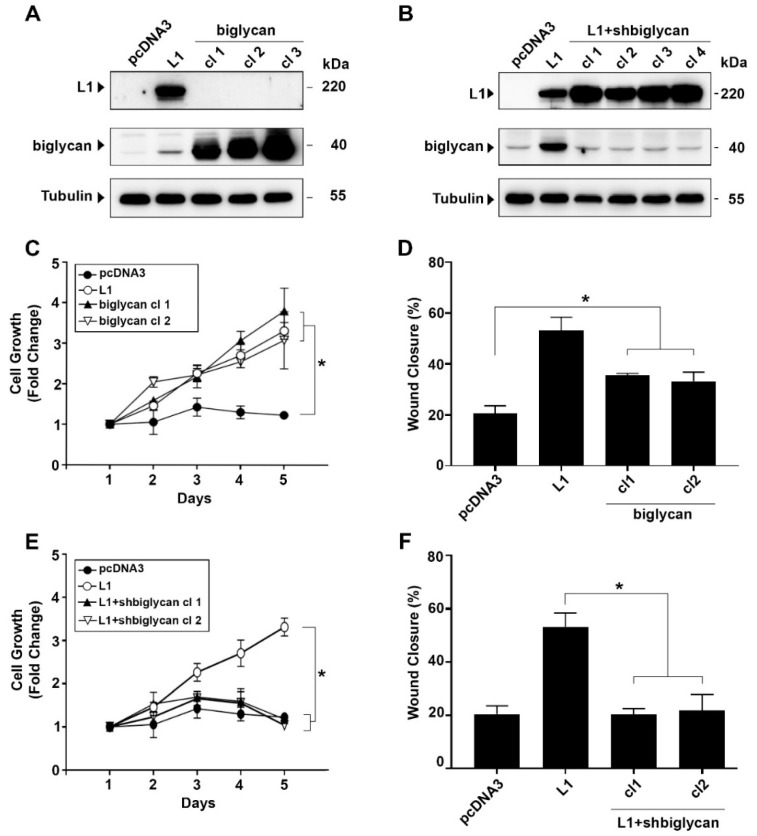
Increased biglycan expression is required for the elevated proliferation and motility of CRC cells expressing L1. (**A**) Individual LS 174T CRC cells clones overexpressing a transfected biglycan cDNA were isolated (cl1, cl2, and cl3), and the level of biglycan in these cells was compared to that of L1-transfected cells (L1) and in cells transfected with an empty pcDNA3 plasmid. (**B**) Expression of endogenous biglycan was suppressed in LS 174T clones transfected with L1 (cl1, cl2, cl3, and cl4) using shRNA sequences that target biglycan (L1+shbiglycan). (**C**) The proliferation of CRC cells expressing L1 (L1), biglycan (biglycan cl1 and cl2), and the empty pcDNA3 plasmid were determined in the presence of 0.1% serum for five days. (**D**) The motility of the CRC cell clones described in (**C**) was determined by the “scratch wound” closure method, 24 h after introducing the wound in a confluent monolayer. (**E**) The proliferation of the CRC cell clones expressing L1 in which the endogenous biglycan levels were suppressed by shRNA (L1+shbiglycan cl1 and cl2) was compared to that of CRC cells expressing L1, or the empty pcDNA3 plasmid. (**F**) The motility of CRC cell cones described in (**E**) was determined by the “scratch wound” closure method, 24 h after introducing the wound. * *p* < 0.05.

To examine the role(s) of changes in biglycan expression on the growth and motile properties of L1-expressing CRC cells, we isolated biglycan-overexpressing CRC cell clones (Figure 2A) and L1-expressing CRC cell clones in which the level of endogenous biglycan was suppressed by shRNA that targets biglycan (Figure 2B). These individually isolated CRC cell clones in which biglycan is overexpressed and CRC cell clones overexpressing L1 in which the endogenous levels of biglycan were suppressed were examined for changes in growth and motility properties. The results shown in Figure 2C demonstrate that the proliferation under stress (in the absence of serum) of CRC cell clones overexpressing biglycan is increased, similar to that observed in cells overexpressing L1. In addition, there was a significant increase in the motile properties of biglycan-overexpressing CRC cell clones (albeit to a lesser extent than in L1-overexpressing cells), as analyzed by the “scratch wound” closure method (Figure 2D). When the level of endogenous biglycan was suppressed in CRC cell clones overexpressing L1, the proliferation (Figure 2E) and motility (Figure 2F) of these CRC cells were reduced to the levels displayed by control LS 174T CRC cells. We conclude that biglycan expression levels play an important role in the L1-mediated increase in CRC cell proliferation and motility.

### 2.3. The L1-Mediated Increase in Cell Proliferation and Liver Metastasis by CRC In Vivo Is Blocked When Biglycan Expression Is Suppressed

**Figure 3:**
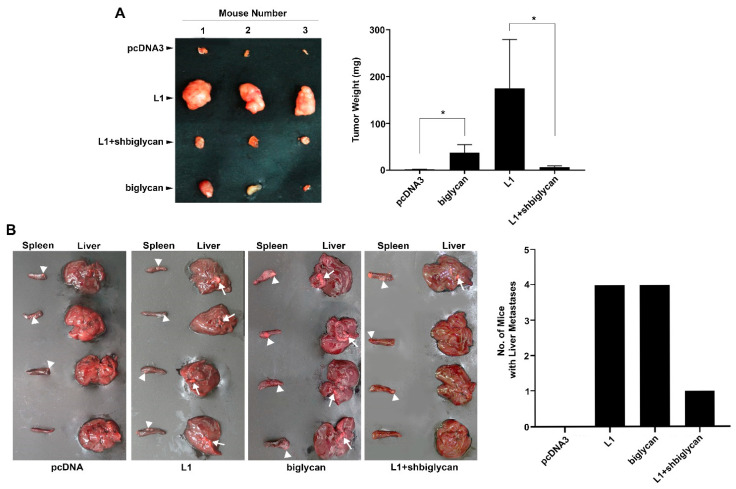
Increasing tumor cell growth and liver metastasis of L1-expressing CRC cells in mice requires biglycan expression. (**A**) The cell lines described in Figure 2A,B were injected subcutaneously into immunocompromised nude mice, and the development of tumors (tumor weight) was determined 2 weeks after injection. (**B**) The cell lines employed in (**A**) were also injected into the tip of the spleen into nude mice, and six weeks after injection, the livers and spleens were excised and photographed. The arrowheads mark tumor development at the injection site in the spleen, while the arrows mark metastases in the liver. * *p* < 0.05.

We wished to determine whether the changes in CRC cell proliferation and motility observed in cultured CRC cells in which biglycan levels were modulated can also be detected in mouse models in vivo. CRC cell clones expressing L1 and CRC cells where the endogenous levels of biglycan are suppressed (see Figure 2B) were injected subcutaneously into immunocompromised nude mice, and the development of tumors was followed for two weeks. As shown in Figure 3A, tumor development by L1-expressing CRC cells was much reduced when the levels of endogenous biglycan were suppressed by shRNA. We have also determined the ability to form liver metastases by these CRC cell clones upon their injection into the spleen, as described [13]. We found that liver metastasis was reduced in L1-expressing CRC cells when the endogenous levels of biglycan were suppressed (Figure 3B, L1+shbiglycan). Biglycan overexpression, on its own, increased cell proliferation and liver metastasis, but was less efficient than L1 overexpression (Figure 3A,B). We concluded that the tumorigenic and metastatic properties conferred by L1 in CRC cells include, as a necessary step, the induction of biglycan expression.

### 2.4. The Induction of Biglycan by L1 Is Mediated by NF-κB Signaling and Requires the Ectodomain of L1

**Figure 4:**
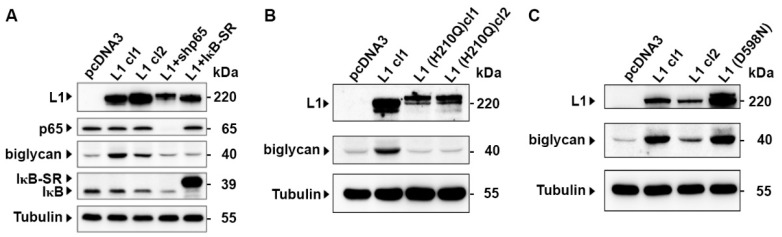
Biglycan induction in L1 expressing cells is blocked when NF-κB signaling is inhibited, or an ectodomain point mutant of L1 is employed. (**A**) The NF-κB pathway was inhibited in L1-expressing CRC cell clones (L1 cl1 and cl2) using an shRNA that targets the p65 subunit of NF-κB (L1+shp65), or the I*κ*B super-repressor (L1+IκB-SR), and the levels of biglycan were determined by Western blot analysis. (**B**) The expression of biglycan was determined by Western blotting in CRC cell clones expressing the ectodomain point mutation in L1 [L1 (H210Q) cl1 and cl2], and (**C**) in the L1 ectodomain point mutant (D598N) and compared to that in pcDNA3 and L1 expressing CRC cell clones. Tubulin was used for monitoring equal protein loading.

Previous studies have shown that the tumorigenic properties conferred by L1 expression in CRC cells require an intact L1 ectodomain that mediates cell-cell adhesion [7] and involves the NF-κB pathway [5,6]. We wished to determine whether the increased expression of biglycan by L1 depends on these characteristics of L1-mediated signaling. The results summarized in Figure 4A demonstrate that when NF-κB signaling was blocked in CRC cells by suppressing the expression of the p65 subunit of NF-κB, using an shRNA to p65, or the IκB super repressor (IκB-SR), the ability of L1 to induce biglycan in L1-expressing CRC cell clones was blocked (Figure 4A). Similarly, CRC cell clones expressing the L1 (H210Q) mutant that cannot confer metastasis [7], do not induce biglycan expression (Figure 4B). In contrast, another mutation in the L1 ectodomain, L1 (D598N), that is suggested to be involved in L1-integrin interactions [7], does not affect the ability of L1 to induce biglycan expression (Figure 4C). Based on these results, we conclude that the induction of biglycan by L1 expression requires an intact L1 ectodomain that regulates L1-L1-mediated adhesion and is transduced via the NF-κB signaling pathway.

### 2.5. Localization of Biglycan Expression in Human CRC Tissue

**Figure 5:**
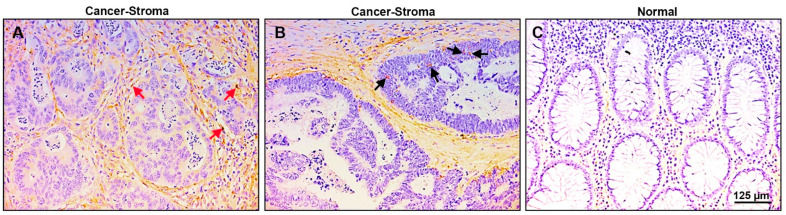
Biglycan is expressed mainly in the stroma of human CRC tissue and is not expressed in normal colonic mucosa. (**A**) Thirty-eight samples from different human CRC tissues were immunostained for biglycan presence. Over 60% of the samples displayed intense staining for biglycan in the stroma around the tumor tissue (red arrows). (**B**) Only sporadic staining of the carcinoma cells was observed (black arrows), and (**C**) no staining for biglycan was detected in the adjacent normal mucosa.

We wished to determine the localization of biglycan in human CRC tissue. We used 38 paraffin-embedded human CRC tissue samples and immune-stained them with anti-biglycan antibodies. The results summarized in Figure 5 show that the tumor tissue displays intense staining for biglycan, mainly in the stromal compartment around the CRC tissue (Figure 5A, red arrows) and only sporadic staining was observed for cancer tissue cells (Figure 5B, black arrows). The normal colonic mucosa did not express detectable levels of biglycan (Figure 5C). Since biglycan is mostly an ECM-associated protein [17], it is not unexpected to see intense staining in the ECM compartment around the tumor, where cancer-associated fibroblasts and infiltrating macrophages and immune cells are present [18]. This robust pattern of biglycan localization in the stroma of CRC tissue suggests that biglycan plays an essential role in human CRC development.

## 3. Discussion

The studies described above demonstrated that we have identified biglycan among the proteins whose levels are highest in the secretome of L1-expressing CRC cells and that the induction of biglycan by L1 is required for CRC progression. We found that suppressing the increase in biglycan in L1-transfected CRC cells blocks the multiple pro-tumorigenic properties conferred by L1 overexpression in CRC cells, including increased proliferation under stress, cell motility, tumorigenesis and liver metastasis in mouse models. Biglycan is a small leucine-rich proteoglycan (SLRP) and is mainly known for its role in the ECM and infiltrating immune cells in the stroma [15,19], which shape the tumor microenvironment. While biglycan is mostly known as a structural element of the ECM, more recent studies have identified critical roles for increased biglycan expression in tumor initiation and progression by playing a role as a signaling molecule in the regulation of inflammation, angiogenesis and autophagy [15,19,20,21]. Moreover, numerous studies have implicated biglycan in contributing to the progression of many types of tumors [16,22], and biglycan is considered a marker for CRC development [16,17,23,24,25,26]. In our immunohistochemical analyses conducted on tissues from CRC patients, high levels of biglycan expression were detected in the stroma around the CRC tissue, which is supportive of the critical role that increased biglycan expression plays in the ECM contributing to tumor progression.

A unique property of L1 expression in CRC cells is that L1 is induced only in invasive CRC cells at the tumor edge but not in the homeostatic colonic mucosa, or even in adenocarcinoma tissue of the colon [2]. Furthermore, our previous studies found that the L1-induced expression of several key genes that participate in L1-mediated signaling requires the ezrin-NF-κB pathway [5,6,14]. In this study, we also identified the NF-κB pathway as a critical regulator of downstream signaling from L1 to biglycan. Interference with NF-κB signaling blocked the induction of biglycan and the pro-tumorigenic properties conferred by L1 in CRC cells. In addition, we identified the L1-ectodomain that regulates L1-L1 adhesion, on the surface of neighboring cells, as an essential mediator of biglycan induction in CRC cells. The identification of biglycan as a key downstream gene of L1-mediated signaling that plays a critical role in CRC invasion is of much importance in our wish to understand L1-mediated CRC progression.

By what mechanism/s is increased biglycan expression in L1-expressing CRC cells contributing to CRC progression? A wide variety of mechanisms have been implicated in biglycan-mediated tumor progression, including upregulation of VEGF [27,28], the binding of biglycan to toll-like receptors [29], or by modulation of signaling pathways including the canonical Wnt pathway [20] and the NF-κB pathway [26]. Future studies will have to determine which of these pathways involving biglycan are responsible for CRC progression in L1-expressing CRC cells.

Taken together, the targeting of biglycan expression in L1-expressing CRC cells promises to be a useful strategy to detect novel therapies of CRC in invasive CRC cells that are positive for L1.

## 4. Materials and Methods

### 4.1. Cell Culture

The LS 174T cell line was cultured in RPMI-1640 (Gibco, Thermo Fisher Scientific, Paisley, UK) supplemented with 10% FBS (Gibco, Thermo Fisher Scientific, Paisley, UK) and 1% penicillin/streptomycin solution (Biological Industries, Beit-Haemek, Israel). LS 174T-L1, LS 174T+biglycan, LS 174T-L1/D598N, and LS 174T-L1/H210Q cells were cultured in RPMI-1640 medium with neomycin (800 µg/mL) [7]. LS174T L1+shbiglycan cells were cultured in RPMI medium-1640 containing both neomycin (800 µg/mL) and puromycin (10 μg/mL) [5,13].

### 4.2. Transfection, Cell Proliferation, and Motility Assays

Transfection of LS174T cells was performed using the Xfect™ transfection reagent (TaKaRa, Mountain View, CA, USA), according to the manufacturer’s instructions. For cell proliferation assays, 2500 cells were seeded in 12-well plates containing RPMI-1640 with 0.1% FBS, and the proliferation rate was assessed by cell counting over a period of 5 days. Cell motility was determined by the artificial “scratch wound” closure assay as described [6].

### 4.3. Plasmids

The biglycan cDNA expression vector was obtained from Dr. Liliana Schaefer (Pharmazentrum Frankfurt/ZAFES, Institut für Allgemeine Pharmakologie und Toxikologie, Klinikum der Goethe-Universität, Frankfurt am Main, Germany) [30]. pSUPER.puro was used to prepare shRNA to biglycan sequences according to the manufacturer’s instructions (pSUPER.puro RNAi System, OligoEngine, Seattle, WA, USA). The target sequences used are described in Supplementary Table S2.

### 4.4. Immunoblotting and Immunofluorescence

The following antibodies were used for immunoblotting: rabbit anti-L1 (provided by Dr. V. Lemmon, University of Miami, Miami, FL, USA) at 1:2000 dilution, mouse anti-biglycan, SC-100857 (Santa Cruz Biotechnology Inc., Dallas, TX, USA) at 1:1000 dilution, rabbit anti-phospho-IκBα #2859 (Cell Signaling Technologies Inc., Danvers, MA, USA) at 1:1000 dilution, rabbit anti-NF-κB p65, sc-109 (Santa Cruz Biotechnology, Inc., Dallas, TX, USA) at 1:1000 dilution, mouse anti-β-tubulin (Sigma-Aldrich, St. Louis, MO, USA) at 1:5000 dilution. RIPA lysis buffer supplemented with 1% complete protease inhibitor cocktail was used to prepare cell lysates as described [7]. The ECL method was used to develop Western blots (Amersham Biosciences, Buckinghamshire, UK).

Immunofluorescence was performed on cells grown on glass coverslips. Cells were permeabilized with 0.5% Triton X-100, fixed with 4% paraformaldehyde [9], incubated with rabbit anti-L1 and mouse anti-biglycan antibodies, both at a 1:200 dilution. Cells were tagged with Alexa Fluor 488-labeled goat anti-mouse IgG (ABCAM, Trumpington, Cambridge, UK) and Cy3-labeled goat anti-rabbit IgG (Jackson Immunoresearch Laboratories, West Grove, PA, USA) secondary antibodies at a dilution of 1:1000. Nuclei were counter-stained with 5 μg/mL 4′-6-diamidino-2-phenylindole (DAPI, Sigma-Aldrich, St. Louis, MO, USA). Images were captured using the Zeiss LSM 800 confocal microscope and ZEN imaging software (Carl Zeiss Microscopy GmbH, Jena, Germany). Analysis of the images was done by Image J and FIJI software.

### 4.5. Quantitative RT-PCR

Total RNA was isolated from cells using the Bio-Tri reagent (Bio-Lab, Jerusalem, Israel) according to the manufacturer’s protocol. Synthesis of first-strand cDNA was carried out using SuperScript™ II Reverse Transcriptase (ThermoFisher Scientific, Waltham, MA, USA) according to the manufacturer’s protocols. The primers used for amplification are described in Supplementary Table S3. Gene expression was determined as fold change in RNA levels that was calculated by the ΔΔCT method using QuantStudio Design and Analysis software v1.5.1 (ThermoFisher Scientific, Waltham, MA, USA).

### 4.6. Tumor Growth and Metastasis Assays

Subcutaneous tumor growth was induced as previously described [4,30]. Briefly, 3.0 × 10^6^ cells in 100 μL PBS were injected at different sites in the flanks of male nude mice [5]. Mice were sacrificed 14 days after injection. Tumor weight was determined, and graphs were plotted using the SigmaPlot statistical analysis software. The ability of cells to metastasize from the spleen to the liver was determined by injecting 3 × 10^6^ cells in 20 μL PBS into the distal tip of the spleen of 4–5-week-old male nude mice as described [4,13]. Mice were anesthetized by peritoneal xylazine and ketamine injection. The animals were sacrificed after six weeks, and primary tumor formation in the spleen and the appearance of metastases in the liver were determined.

### 4.7. Ethics Approval

The Weizmann Institutional Animal Care and Use (IACUC) ethics committee reviewed, approved, and supervised the animal studies.

### 4.8. Immunohistochemistry

Immunohistochemistry was performed on 38 paraffin-embedded human colorectal adenocarcinomas using a rabbit polyclonal anti-biglycan antibody (NBP1-84971, Novus Biol., Centennial, CO, USA), at a dilution of 1:650, as previously described [2].

### 4.9. Statistical Analysis

Statistical analysis was performed on SigmaPlot v11 software and the significance of results was calculated using the student’s non-paired *t*-test. A *p*-value of <0.05 was considered significant and marked by an asterisk.

## Data Availability

Not Applicable.

## Supplementary Materials

The following supporting information can be downloaded at: https://www.mdpi.com/article/10.3390/ijms23010445/s1.
