# Supplementary material for: A Necessary Role for Increased Biglycan Expression during L1-Mediated Colon Cancer Progression
Source: Int J Mol Sci. 2021 Dec 31;23(1):445. doi: 10.3390/ijms23010445 (PMC8745639; doi:10.3390/ijms23010445)
Supplement: Supplementary file 1 [file ijms-23-00445-s001.zip › ijms-1540663-supplementary.pdf]

Supplementary Materials

A necessary role for increased biglycan expression during L1-mediated colon cancer progression

Arka Saha [1*], Sanith Cheriyamundath [1*], Anmol Kumar [1], Nancy Gavert [1], Thomas Brabletz [2] and Avri Ben-Ze'ev [1#]

[1] Department of Molecular Cell Biology, Weizmann Institute of Science, Rehovot 7610001, Israel; arka.saha@weizmann.ac.il (A.S.); sanith.cheriyamundath@weizmann.ac.il (S.C.); anmol.kumar@atmiyauni.ac.in (A.K.); nancy.gavert@weizmann.ac.il (N.G.)

[2] Experimental Medicine I, Nikolaus-Feibiger-Center for Molecular Medicine, University of Erlangen-Nuernberg, Erlangen 91054, Germany; thomas.brabletz@fau.de

# Correspondence: avri.ben-zeev@weizmann.ac.il

* Equal contribution

Table S1: List of proteins secreted at higher level from LS174T cells expressing L1 compared to control LS174T cells

Gene Symbol	Description	Fold Change
CALCA	Calcitonin-related polypeptide alpha	400
HADHB	Hydroxyacyl-CoA dehydrogenase trifunctional multienzyme complex beta	382.6
MUC2	Mucin 2	82.6
BGN	biglycan	68
SMOC2	SPARC related modular calcium binding protein 2	53
CTSD	Cathepsin D	30.3
VCAN	Versican	18.1
SEMA3B	Semaphorin 3B	15.5
ADPRHL2	ADP-ribosylhydrolase like 2	11

Mass spectrometric analysis of proteins secreted into the cell culture medium from LS174T cells stably expressing L1 compared to LS174T cells transfected with pcDNA3. Proteins whose level was increased >10 fold in L1-expressing cells are shown.

Table S2: Sequences of shRNA targeted against biglycan RNA

Name	Primer Sequence
shbiglycan-1	GATCCCCGAGAACAGTGGCTTTGAACTTCAAGAGAGTTCAAAGCCACTGTTCTCTTTTTA
shbiglycan-2	GATCCCCCCAGATCAGGATGATCGAGTTCAAGAGACTCGATCATCCTGATCTGGTTTTTA
shbiglycan-3	GATCCCCGCTCAACTACCTGCGCATCTTCAAGAGAGATGCGCAGGTAGTTGAGCTTTTTA
shbiglycan-4	GATCCCCATCCAGGCCATCGAACTGGTTCAAGAGACCAGTTCGATGGCCTGGATTTTTTA

Table S3: Primers used for qRT-PCR experiment

Gene	Forward	Reverse
biglycan	TTTGAGCAGAGAGGCTTCTGG	AAAGGACACATGGCGCTGTAG
L1	TCGCCCTATGTCCACTACACCT	ATCCACAGGGTTCTTCTCTGGG
GAPDH	GTCTCCTCTGACTTCAACAGCG	ACCACCCTGTTGCTGTAGCCAA
